# Evaluation of transfer ensemble learning-based convolutional neural network models for the identification of chronic gingivitis from oral photographs

**DOI:** 10.1186/s12903-024-04460-x

**Published:** 2024-07-17

**Authors:** Wen Li, Enting Guo, Hong Zhao, Yuyang Li, Leiying Miao, Chao Liu, Weibin Sun

**Affiliations:** 1 Department of Cariology and Endodontics, Nanjing Stomatological Hospital, Affiliated Hospital of Medical School, Research Institute of Stomatology, Nanjing University, Nanjing, China; 2 Division of Computer Science, The University of Aizu, Aizu, Japan; 3 Department of Orthodontic, Nanjing Stomatological Hospital, Affiliated Hospital of Medical School, Research Institute of Stomatology, Nanjing University, Nanjing, China; 4 Department of Periodontics, Nanjing Stomatological Hospital, Affiliated Hospital of Medical School, Research Institute of Stomatology, Nanjing University, Nanjing, China

**Keywords:** ConvNet models, Ensemble learning, Transfer learning, Gingivitis

## Abstract

**Background:**

To evaluate the performances of several advanced deep convolutional neural network models (*AlexNet*, *VGG*, *GoogLeNet*, *ResNet*) based on ensemble learning for recognizing chronic gingivitis from screening oral images.

**Methods:**

A total of 683 intraoral clinical images acquired from 134 volunteers were used to construct the database and evaluate the models. Four deep ConvNet models were developed using ensemble learning and outperformed a single model. The performances of the different models were evaluated by comparing the accuracy and sensitivity for recognizing the existence of gingivitis from intraoral images.

**Results:**

The *ResNet* model achieved an area under the curve (AUC) value of 97%, while the AUC values for the *GoogLeNet*, *AlexNet*, and *VGG* models were 94%, 92%, and 89%, respectively. Although the *ResNet* and *GoogLeNet* models performed best in classifying gingivitis from images, the sensitivity outcomes were not significantly different among the *ResNet*, *GoogLeNet*, and *Alexnet* models (*p*>0.05). However, the sensitivity of the *VGGNet* model differed significantly from those of the other models (*p* < 0.001).

**Conclusion:**

The *ResNet* and *GoogLeNet* models show promise for identifying chronic gingivitis from images. These models can help doctors diagnose periodontal diseases efficiently or based on self-examination of the oral cavity by patients.

## Background

Gingivitis is a chronic disease primarily caused by bacterial infection. Gingivitis affects public health worldwide, and its main clinical symptoms are bleeding, redness, and bad breath. Without prompt treatment, the continuous progression of gingivitis can lead to the resorption of the alveolar bone and loss of the periodontal ligament. Meanwhile, the latest epidemiological survey on oral health reported that 88% of adults suffer from gingivitis each year [1]. As the first stage of periodontal disease, gingivitis is closely related to other serious diseases such as cardiovascular disease, leukemia, and tumors [2, 3]. Protecting gum health is a key factor in preventing periodontal disease [4]. Gingivitis is clinically diagnosed through a series of conventional oral examinations, which include measurements of the degree of redness and swelling and bleeding on probing [5]. Although chairside oral examination is the most reliable method for gingivitis detection, other methods should be considered to improve the efficiency of diagnosis and reduce the clinical burden on physicians.

In recent years, deep learning algorithms (e.g., deep convolutional neural networks [DCNNs]) have shown high efficiency and accuracy for classifying and analyzing medical characteristics or features [6, 7]. These algorithms can enable the automatic screening of several diseases using imagery captured with cameras or smartphones. For example, Li et al. classified tooth types in dental images using contrast-limited adaptive histogram equalization, gray-level co-occurrence matrix, and extreme learning machine approaches [8]. Krois et al. applied CNNs to detect periodontal bone loss in panoramic dental radiographs [9]. In a previous study, we screened for gingivitis and its irritants (dental calculus and soft deposits) in oral photos using a novel multi-task learning CNN model and obtained good accuracy and sensitivity for both classification and localization [10]. The above examples demonstrate that image analysis can play an important role in monitoring the gingival condition of patients, and that DCNNs show promise for supplementing clinical visits to detect health issues. However, using deep learning models to screen for gum conditions remains under-explored.

In this study, we selected several advanced deep ConvNet models (*Alexnet* [11], *VGG* [12], *GoogLeNet* [13], and *ResNet* [14]) for the classification of anatomical gingival soft tissue structures from oral screening images and compared their performance for recognizing chronic gingivitis in terms of both accuracy and efficiency. AlexNet is renowned for its breakthrough performance in the ImageNet challenge, which reignited the interest in neural networks for computer vision tasks. GoogLeNet, with its inception modules, demonstrates the power of network-in-network architectures to increase depth and width without a significant increase in computational cost. ResNet introduced residual learning, enabling the training of much deeper networks by addressing the vanishing gradient problem, leading to remarkable improvements in accuracy. Lastly, VGGNet is celebrated for its simplicity and depth, utilizing very small convolutional filters to build deeper networks, which has shown to be effective in capturing fine details in images. Together, these models encompass a range of approaches that have significantly advanced the field of computer vision. By comparing the accuracy of the four advanced models in identifying gingivitis, we aim to initiate a discourse on the integration of deep learning into oral self-examination tools for enhancing public dental health.

## Materials and methods

In this section, Firstly, we present the data collection protocol employed in this study along with the generated dataset, followed by the annotation of the collected data. Next, we demonstrate the limitations associated with gingivitis diagnosis and propose four deep learning model architectures. The implementation and training of the model are subsequently elaborated upon. Finally, we elucidate the metrics and statistical analysis methodologies employed to validate multiple models for discerning gingivitis.

### Ethical approval

The project was approved by the Ethical Review Board at local University (approval number NJSH-2022NL-069). Any reports related to the results of this study will be subject to confidentiality and compliance with data protection regulations

### Subjects and dataset

**Fig. 1:**
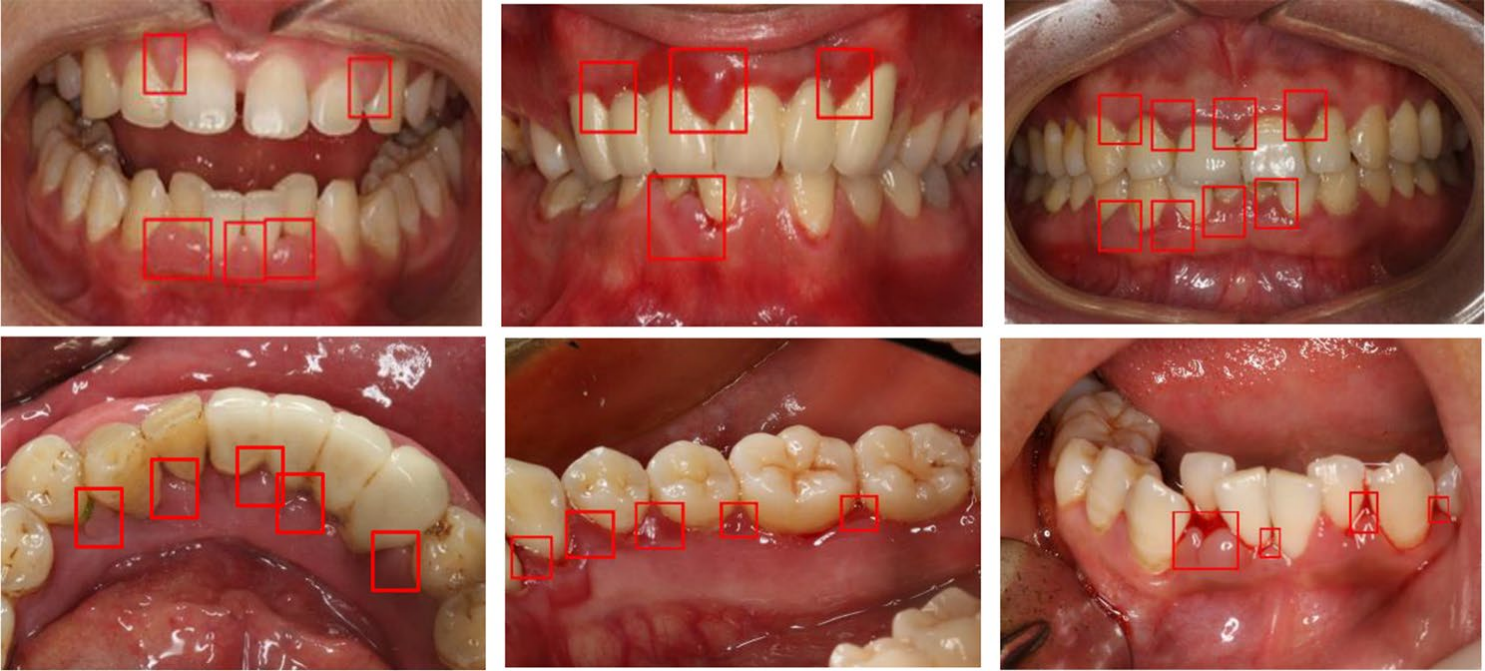
Clinical symptoms of gingivitis

We built an in-house dataset of oral photos collected in the Department of Periodontics, Orthodontics and Endodontics, Stomatological Hospital, from January 2020 to December 2022. The dataset contained 683 images captured by postgraduate dentists from 134 gingivitis patients and the healthy population. The images cover a wide age range from 14 to 60 years old. Images of teeth with severe cervical caries and periodontitis with severe gingival recession were excluded. To approximate the image quality in practical scenarios, a diverse range of equipment was utilized for photo collection, including iPhone, Samsung Galaxy, Canon 6D and so on. The methods were conducted in accordance with the relevant guidelines and regulations, written informed consent was obtained from each participant. The diagnosis of chronic gingivitis requires that two criteria be met: (1) clinical symptoms including bleeding with tooth brushing, blood in the saliva, and gingival swelling and redness (Fig. 1); and (2) no attachment loss in periodontal probe examination and no loss of supporting structures in radiographic analysis.

In our preprocessing step, images of different sizes are first scaled, and all images are uniformly scaled to a size of 224 × 224 pixels. This process uses bilinear interpolation to maintain the aspect ratio and detail information of the image. Subsequently, we normalized the scaled image to normalize the pixel values to between 0 and 1.

Given the constrained size of our dataset, it was imperative to explore strategies that could artificially expand our data’s diversity without compromising its integrity. To this end, we integrated a comprehensive suite of data augmentation techniques directly into our training process. This dynamic augmentation occurs in real-time during the training phase, presenting the model with an enriched dataset. Our augmentation strategy includes a range of transformations, such as random rotations zooming, horizontal flipping, and shear transformations.

**Fig. 2:**
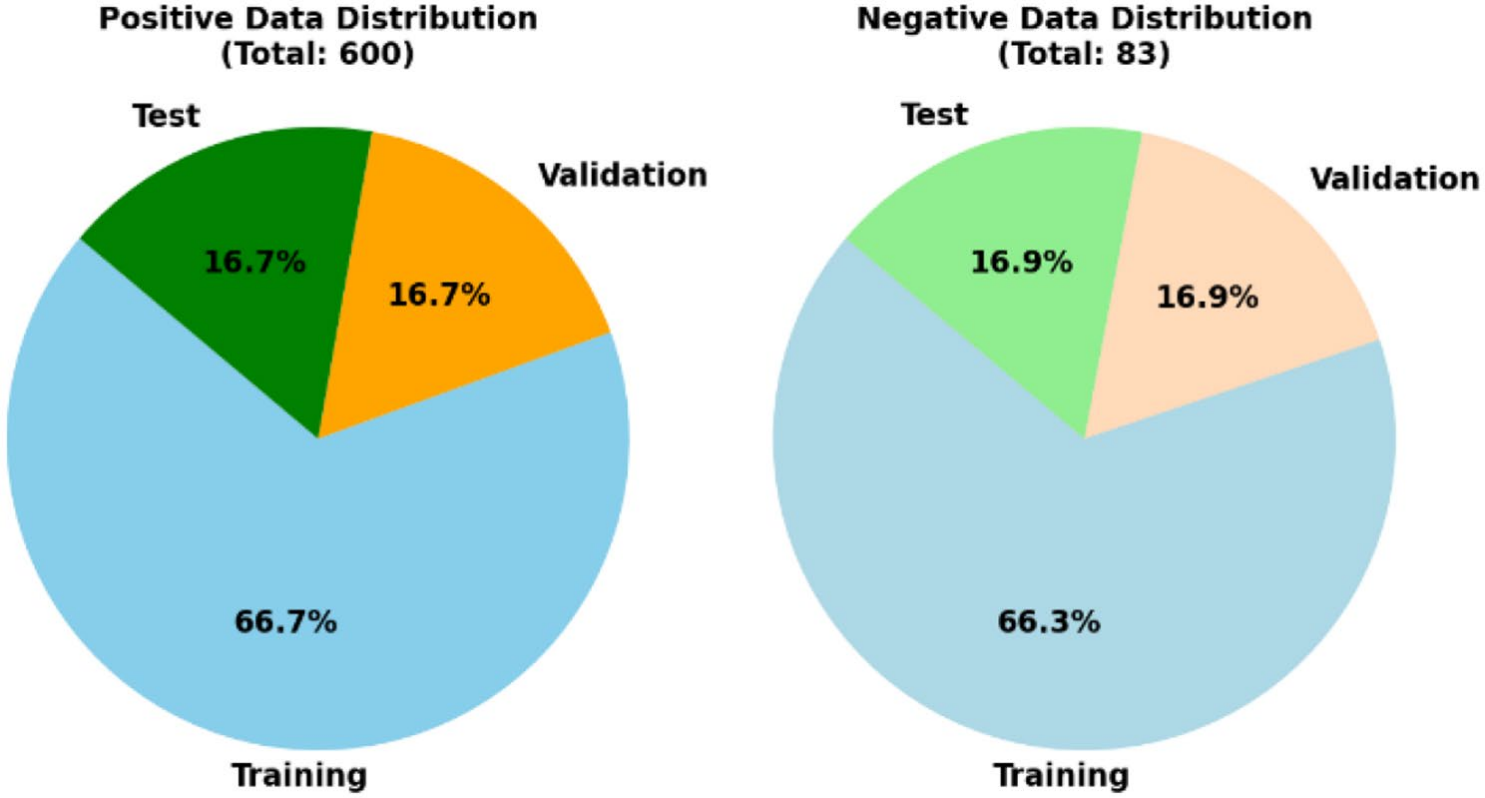
Numbers of images with positive and negative findings assigned to the training, validation, and testing subsets

Implicit sorting relationships may exist in the initially gathered data, which may negatively affect the DCNN model’s accuracy. Therefore, during training, we initially use a shuffling procedure to disrupt the order of the data.We divided the dataset into training, validation, and testing subsets by randomly splitting the photos into three groups. We used the training set to update the model. We changed the hyperparameters in the validation set, and the test set was used to evaluate the model’s performance. To increase the use of data and train the model with a large number of parameters, we improved the efficiency of data utilization through cross validation. Figure 2 provides details about the distribution of the dataset.

#### 1) Training dataset

The identification of gingivitis is a complex process that involves considering the color of the gums, the level of swelling, and the bleeding condition. Accordingly, we selected models with large numbers of structural layers to extract the complex features of gingivitis. To update the model’s parameters, the model was trained many times with the training data.

#### 2) Validation dataset

The model’s variables, including the number of layers and neurons, can affect the ultimate recognition accuracy. In this study, the model that performed best in the validation set was chosen as the final version after we tested numerous iterations of each model. As the number of training sessions increases, the model accumulates considerable useless knowledge, including the brightness of the image, the placement of the teeth, the size of the teeth, and other features of oral diseases such as black stains and dental calculus. Additionally, we used the validation set to halt model updates early.

#### 3) Test dataset

We used the test set to test the final performance of the model based on accuracy and analysis of the receiver operating characteristic (ROC) curves.

#### 4) Cross-validation method

**Fig. 3:**
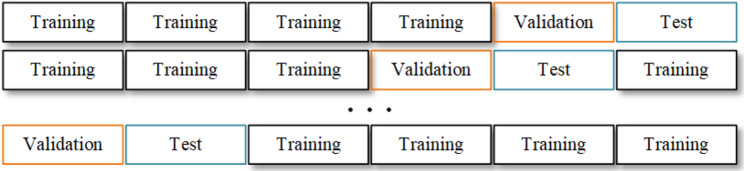
Cross validation was used to average the data and take several samples to observe the results of a variety of collected data

Due to the complexity of the task and the small amount of data, we needed to increase the use of the collected data. We used a cross-validation method to train/test multiple groups of models with different training/test sets, as shown in Fig. 3.

This rigorous validation framework was designed to enhance the reliability of our model’s performance metrics by carefully segregating the dataset into non-overlapping training, validation, and testing segments. The nested cross-validation process involved an outer 5-fold cross-validation for delineating training and testing data, complemented by an inner 5-fold cross-validation within the training dataset for hyperparameter tuning.

### ConvNet models

**Fig. 4:**
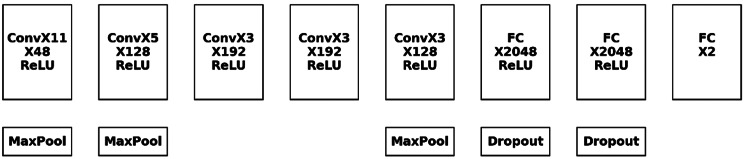
Architecture of AlexNet, *Note* “Conv” is short for convolutional, and “FC” stands for the fully connected layer

We selected several models and compared their performances by training to identify the model with the best performance. Finally, we outperformed a single model by using ensemble learning. Figure 4 shows the overall framework of the model.

#### 1) AlexNet

**Fig. 5:**
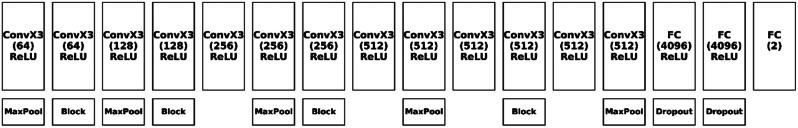
Architecture of VGG. *Note* “Conv” is short for convolutional, and “FC” stands for the fully connected layer

*AlexNet* uses ReLU as the activation function. Only the most obvious elements of a region are kept. As an illustration, consider the color of the gingiva. Only the components whose color depth exceed the threshold are kept, and the rest of the features are eliminated (Fig. 5).

To increase the diversity of the data, we randomly eliminated some of the intermediate results using the dropout function.

#### 2) VGG

**Fig. 6:**
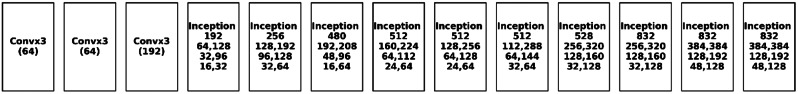
Architecture of GoogleNet. *Note* “Conv” is short for convolutional. Inception is a structure composed of 5 convolutional networks, where the dimensions of each network are as shown in the figure

For the model to quickly collect the general information of the image, a lot of distant information needs to be swiftly aggregated. We used the 3 × 3 convolution kernel to quickly gather tooth and gingival information over large distances. Therefore, we obtained a general assessment without a precise division of tooth position (Fig. 6).

#### 3) GoogLeNet

**Fig. 7:**
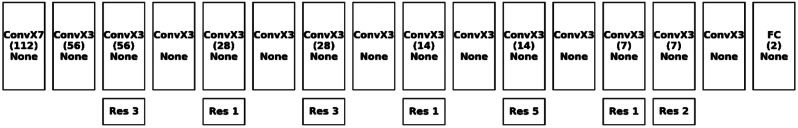
Architecture of ResNet. *Note*: “Conv” is short for convolutional, “Res” is short for Residual Network, and “FC” stands for the fully connected layer

Each model layer’s dimension may correlate to the tooth’s higher-dimensional attributes. To minimize the feature’s dimension, we used a 1 × 1 convolution kernel (Fig. 7).

#### 4) ResNet

**Fig. 8:**
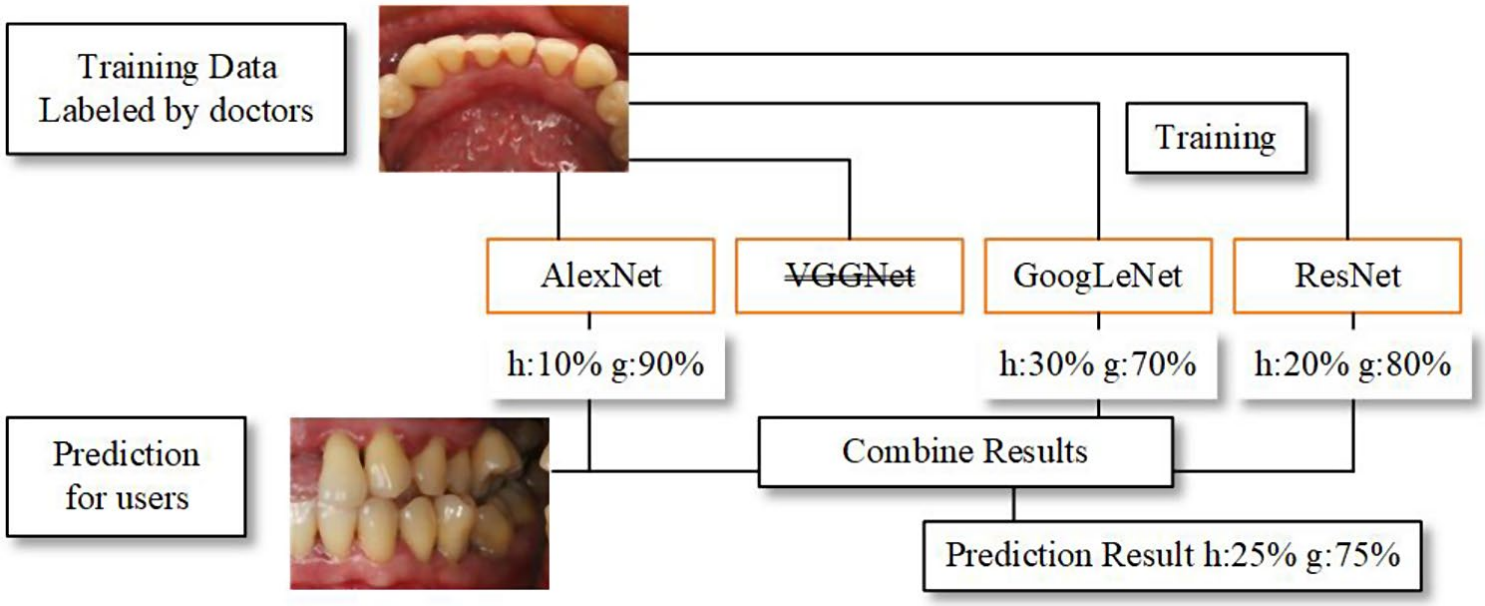
We used ensemble learning to identify images that are difficult for a single model to identify

We used a 34-layer version of the ResNet model (Fig. 8). We discovered that the starting information and position information in the image were helpful in the recognition process. Basic knowledge of the structure and color can be learned in the first few layers, and this knowledge remains helpful in the subsequent layers. ResNet adds the results of previous layers to subsequent layers by using residual structures and skipping intermediate steps. In this way, the knowledge learned in the previous layer can be directly transferred to the next layer.

#### 5) Ensemble learning

The *AlexNet*, *GoogLeNet*, and *ResNet* models had good performance based on their accuracy and area under the ROC curve (AUC). In contrast, the *VGGNet* model performed poorly. We know that for locating particular data, some models perform poorly, while others show good performance. Consequently, we combined the findings from the three models with good performance using ensemble learning. Three models—*Alexnet, Googlenet, and Resnet*—were used to make predictions for one image. The output of ensemble learning was then determined by the outcomes of the three models with the highest number. The ensemble learning process is shown in Fig. 4.

### Training strategies

To train the model quickly, we employed transfer learning hyperparameters. The optimization method included a few hyperparameters. Transfer learning [15] was first utilized for initialization. The model parameters developed using the open dataset were utilized by all four models. Compared with the loss value of the model, which will decrease later, the loss value of the model in the early stage of training is larger. The early loss value of training was greater because the hyperparameters used in transfer learning were different from those employed in our jobs. The GoogLeNet and ResNet models both showed substantial loss values in the first few epochs, whereas the VGG and AlexNet models displayed smaller loss values. The loss values of the four prediction algorithms converged to lower values as the number of epochs increased [16].

In our endeavor to develop a model capable of generalizing well beyond the confines of our training data, we implemented a comprehensive strategy to prevent overfitting. Apart from leveraging early stopping as a safeguard against overtraining, we incorporated dropout with a rate of 0.5 within our model’s architecture, which served to randomly deactivate a portion of the neurons during each training pass. This randomness introduced by dropout helps in reducing the model’s sensitivity to specific features of the training data. In parallel, L2 regularization with a value of 1 × 10^− 4^ was applied across the network’s parameters, imposing a constraint that penalizes the magnitude of the weights, thereby discouraging complex models that could overfit the training data.

To evaluate the effectiveness of the proposed strategy, we ran numerous experiments. Inference accuracy and training loss were measured. Finally, we examined the performances the *AlexNet*, *VGG*, *GoogLeNet*, and *ResNet* deep learning models.

**Table 1:** The precision, recall, F1 score, cross entropy loss and accuracy of models

	Precision	Recall	F1_score	Cross Entropy	Accuracy
AlexNet	0.98	0.92	0.95	0.27	92%
GoogLeNet	0.98	0.91	0.93	0.32	90%
ResNet	0.97	0.87	0.92	0.38	87%
VGGNet	0.97	0.85	0.90	0.42	85%

In our study, we evaluated several deep learning models’ performance metrics, including precision, recall, F1 score, cross-entropy loss, and accuracy, as summarized in Table 1. AlexNet and GoogLeNet demonstrated superior performance, particularly in precision and F1 score, indicating their effectiveness in accurately identifying positive cases.

The convergence performance of a model is reflected by the loss value. In most cases, the loss value will stabilize after a certain time and eventually decrease to some amount. The training is then considered to be finished. The model’s training outcome can be measured by the accuracy. The initial prediction accuracy of the *AlexNet*, *VGG*, and *Google*Net models was roughly 85%. The *VGG* model began to exhibit accuracy fluctuations at approximately 20 epochs, after which the accuracy increased steadily before flattening out. The same phenomenon was observed for the *AlexNet* model at approximately 23 epochs. At approximately 29 epochs, the accuracy of the *AlexNet* model peaked before starting to decrease and returning to the initial accuracy.

A recognition model is prone overfitting if there are too many training iterations, which increase the accuracy on the training dataset. However, this will result in substandard generalization performance, leading to poor performance on unseen data. Among the four models, the *ResNet* and *GoogLeNet* models performed well with high recognition accuracy. We also constructed the ROC curves of the four models and calculated the corresponding AUC values. Among the models, VGGNet had the smallest AUC (89%), while the AUCs of *GoogLeNet* and *ResNet* were larger (94% and 97%, respectively). It is worth noting that *GoogLeNet* showed better generalization performance than *ResNet*.

It is important to note that after sufficient training, several models experienced overfitting. As a result, we kept the results once the training reached the highest level of accuracy.

We examined the *p* values between different models and correct result after extracting the data at random. *ResNet* and *GoogLeNet* and label(The label is the real classification of the data. During the process of data collection, the researchers who collect the data will mark the data classification as the label.)had the highest *p* values out of all the tested models, and no substantial differences were observed in the projected outcomes of these models. The *p* values between the *VGGNet* model and the other models were all less than 0.001, indicating significant differences.

**Table 2:** Calculated *p* values between different models and labels after extracting the data at random

	Label	AlexNet	GoogLeNet	ResNet	VGGNet
Label		0.164	0.856	0.908	< 0.001
AlexNet	0.164		0.226	0.201	< 0.001
GoogLeNet	0.856	0.226		0.948	< 0.001
ResNet	0.908	0.201	0.948		< 0.001
VGGNet	< 0.001	< 0.001	< 0.001	< 0.001	

## Discussion

Chronic gingivitis is a common oral disease that influences human health and quality of life [17]. In this study, we evaluated four precise ConvNet models for the recognition of chronic gingivitis from screening oral photographs taken by either mobile phones or digital cameras. This approach differs from the commonly used deep learning systems based on clinical imaging for the computer-assisted diagnosis of gingivitis. Among the four tested models, the *ResNet* and *GoogLeNet* models presented the best performance. These models can be applied to help people maintain oral hygiene through self-screening, thereby reducing the treatment burden on physicians and the financial burden on patients.

Previous studies have verified the advantages of using photographs for the detection of chronic gingivitis by dentists. Eke et al. reported self-screening measures for periodontal diseases [18]. Alalharith et al. evaluated state-of-the-art object detection and recognition techniques based on deep learning for the automatic detection of gingivitis in orthodontic patients based on intraoral images [19]. Lang et al. further assessed the ability to diagnose plaque-induced gingivitis from intraoral photographs based on the symptoms of gingivitis [20]. These studies all suggest that clinical photographs are sufficient for dentists to draw conclusions that are statistically similar to those based on visual inspection.

Unlike the above-described studies, we compared several advanced transfer learning-based ConvNet models (*Alexnet*, *VGG*, *GoogLeNet*, and *ResNet*) for the automated detection of gingivitis from oral photographs. The most closely related study in the literature is Xianwei et al. (2019), who assessed a deep learning method (a mask DCNN model with a multichannel gray-level co-occurrence matrix associated with a particle swarm optimization neural network) for the detection of gingivitis based on dental images. However, compared to their work, our study provides four innovations: (1) we identified gingivitis using oral images from both consumer mobile phones and digital cameras; (2) we employed transfer learning to effectively train the models and compensate for the low data volume; (3) we used a cross-validation method to process the training, validation, and testing datasets, and each group was trained once to make efficient use of the datasets; and (4) we use ensemble learning, a supervised learning algorithms, to select three models that performed better than *VGGNet*.

At present, transfer learning has three main aspects [21]: (1) the new data are used for training after loading all the model parameters; (2) only the last few layers of parameters are trained and loaded after the weights are determined; and (3) after the weight is loaded, a complete connection adds one layer to the original network. Because our task is not the same as the publicly available dataset, we adopted transfer learning and trained the model faster than random initialization.

Other studies have used transfer learning methods to diagnose oral diseases. Jin et al [22]. proposed a deep transfer learning method for the diagnosis of various diseases based on computer-aided facial recognition. Rahman et al. [23]. proposed a transfer learning model based on *AlexNet* to extract rank features from oral squamous cell carcinoma biopsy images and achieved high classification accuracies. Chang et al. [24]. presented a transfer learning-based method for the automatic diagnosis of parotid gland tumors from multimodal magnetic resonance images. However, none of these studies used ensemble learning to train for low-quality spatial annotations of disease.

**Fig. 9:**
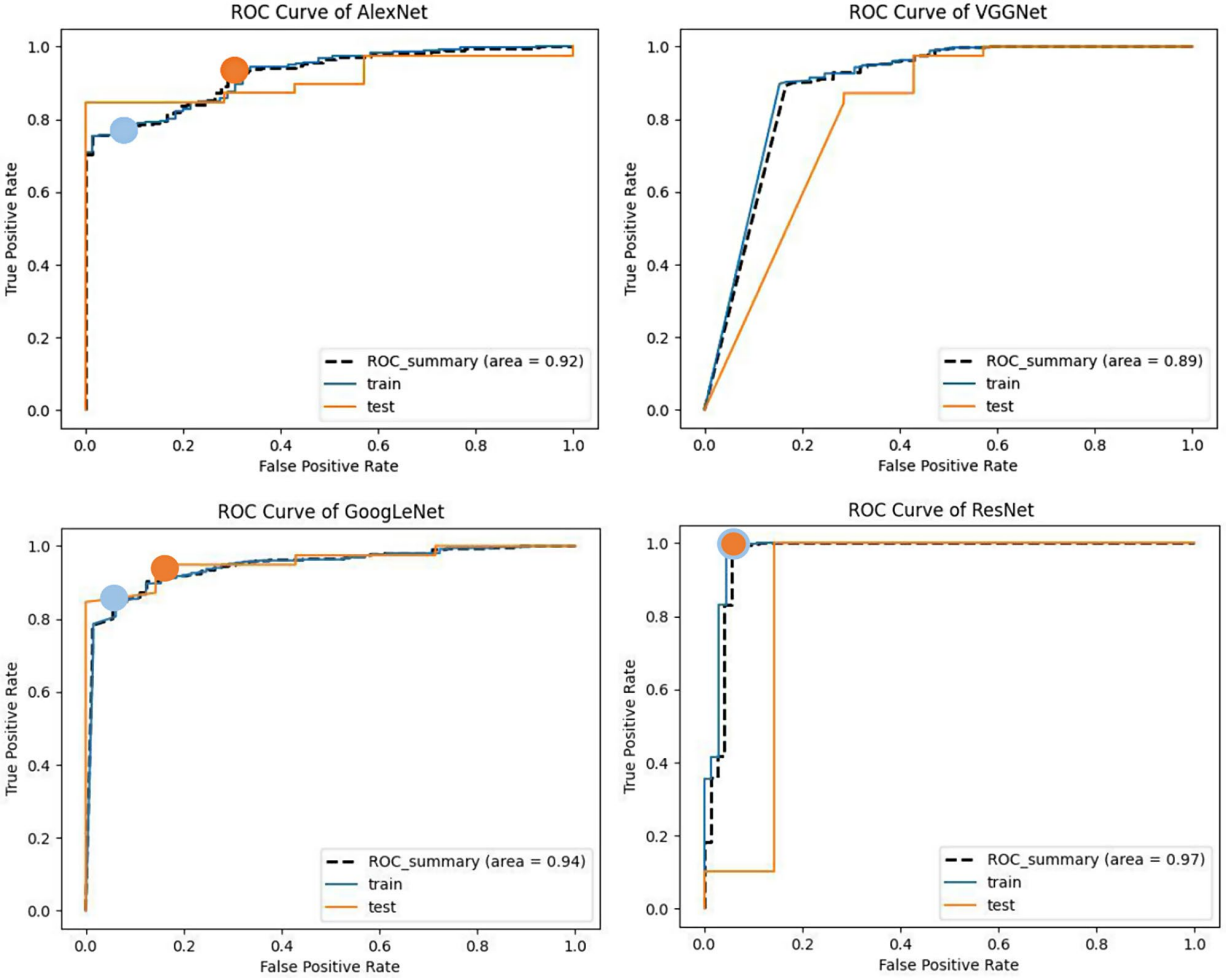
ROC curves of *AlexNet*, *VGG*, *GoogLeNet*, and *ResNet* during training. To reduce false positives, we established a high-specificity point (blue) with a high discrimination threshold. To maintain a low missing rate, we selected a high-sensitivity point (orange) with a low discriminating threshold. These points were not simultaneously established for the *VGGNet* model since its prediction result was suboptimal

**Fig. 10:**
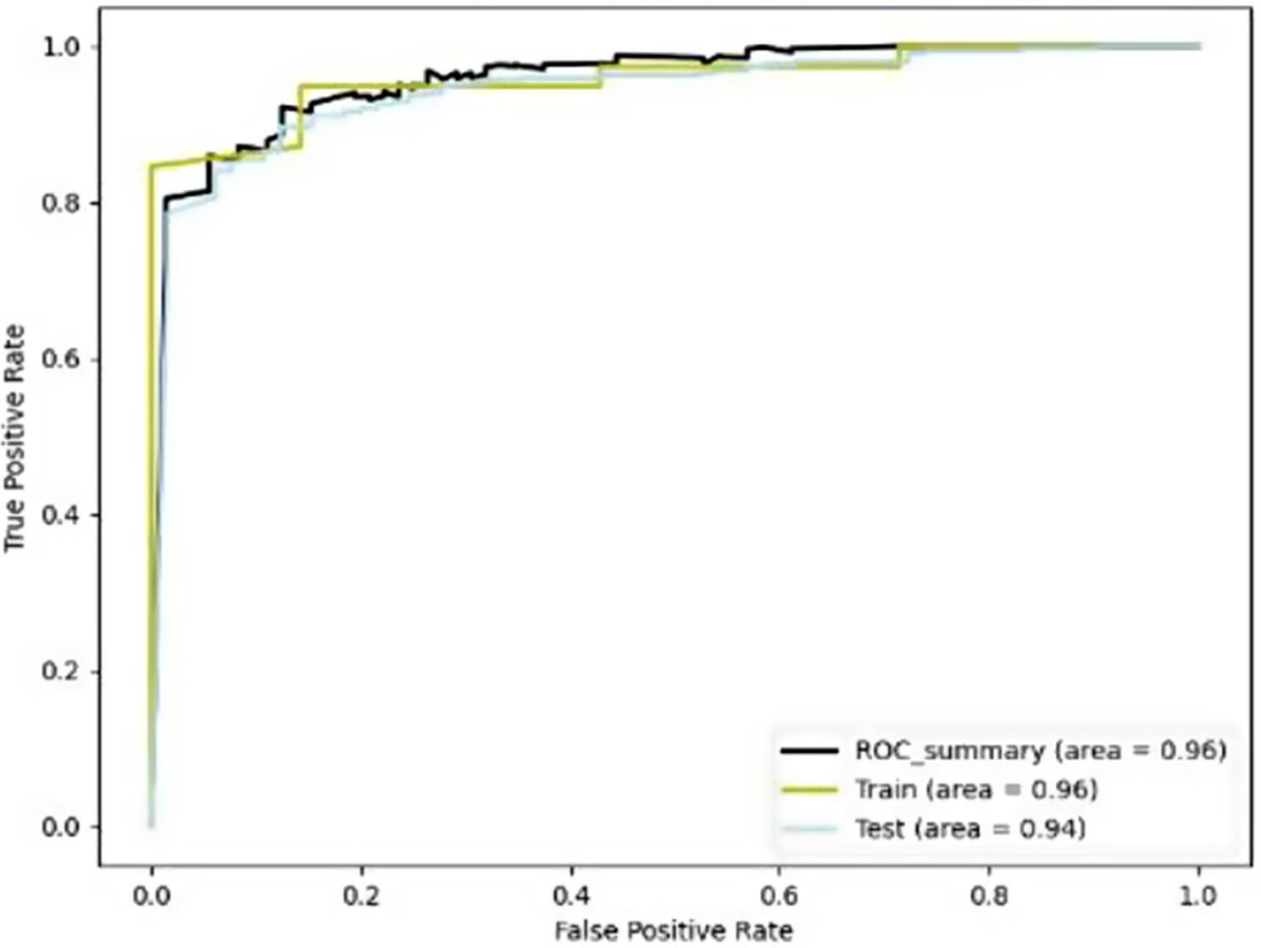
ROC curves with ensemble Model

**Fig. 11:**
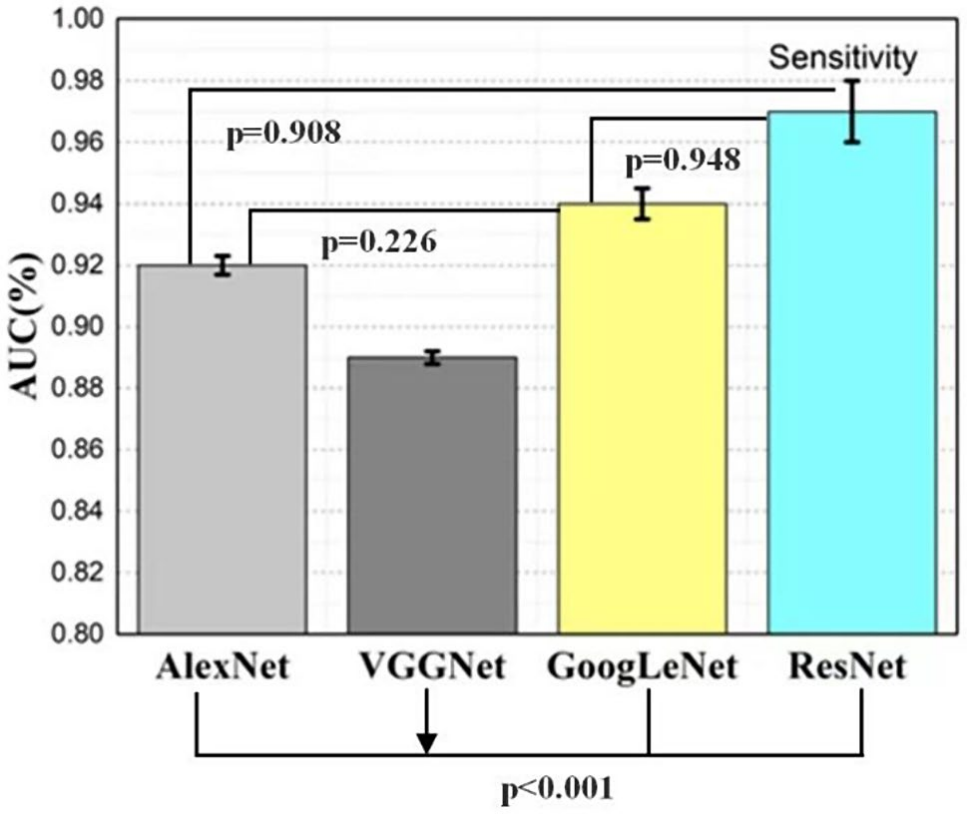
Sensitivity of the *AlexNet*, *VGG*, *GoogLeNet*, and *ResNet* models during training and testing. The AUC value increased during training and decreased during testing. Among the models, the prediction result of *ResNet* changed the most

In this work, we developed four trained CNN models to automatically identify and localize gingivitis from photographs. We created a high-specificity point (blue) and a high-sensitivity point (orange) for the model to show the accuracy; as a result, the prediction results at various operating points could be represented visually. To reduce false positives, we established a high-specificity point (blue) with a high discrimination threshold. To maintain a low missing rate, we selected a high-sensitivity point (orange) with a low discriminating threshold (Fig. 9). We also provide the ROC curve or accuracy of the ensemble model in Fig. 10. It is important to note that the two points of *ResNet* can be obtained at the same site. These points were not simultaneously established for *VGG*Net since its prediction result was suboptimal. Regarding their ability to recognize gingivitis, the *ResNet* model achieved a larger AUC of 97% than the *GoogLeNet* (AUC = 94%), *Alexnet* (AUC = 92%), and *VGG* (AUC = 89%) models (Fig. 9). The *ResNet* and *GoogLeNet* models and label (correct category result) had the highest sensitivity of all models tested, and there was not a substantial difference in the projected outcomes between the *ResNet* and *GoogLeNet* groups (*p*>0.05). Meanwhile, the sensitivity of *VGGNet* was significantly different from those of the other groups (*p* < 0.001; Table 2; Fig. 11).

**Fig. 12:**
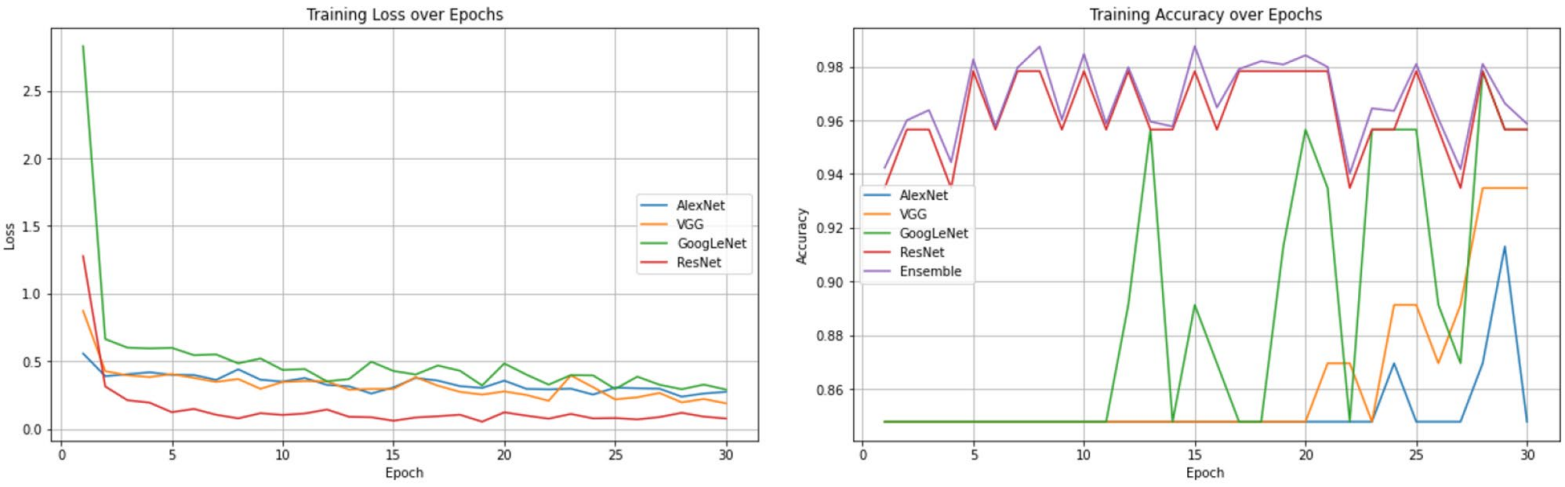
Loss and accuracy of the *AlexNet*, *VGG*, *GoogLeNet*, and *ResNet* models during training

**Fig. 13:**
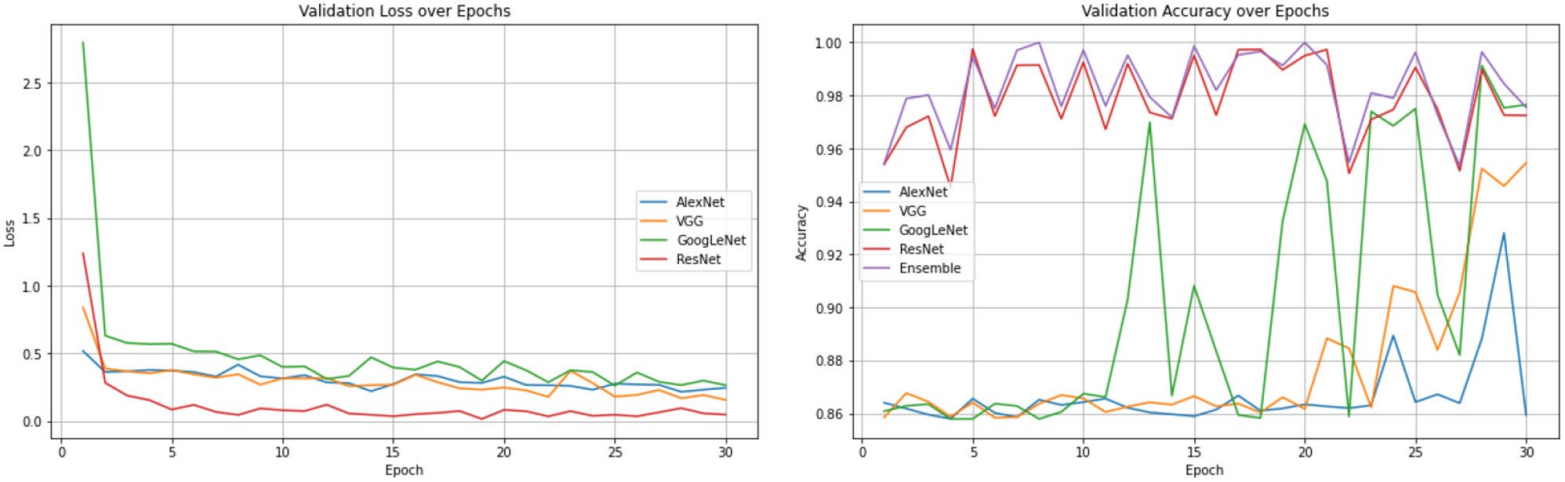
Loss and accuracy of the *AlexNet*, *VGG*, *GoogLeNet*, and *ResNet* models of Validation

The task addressed in this study was a dichotomous classification task, and the amount of data was less than required for model training datasets. To make use of this small amount of data and ensure the accuracy of the model training process, we employed transfer learning during training. Well-learned networks retain some common abstractions of gingivitis, and the new neural network can adopt the high-latitude features of the previous underlying general-purpose network. Transfer learning can hasten the convergence of the model and enhance recognition performance. The loss of GoogleNet and ResNet models continued to fluctuate until 25 epochs, after which they stabilized (Figs. 12 and 13). After a few variations, Alexnet and VGG quickly stabilized after roughly the same number of epochs. It is important to note that the model’s prediction performance is shown by the accuracy on the right. We chose the best result as the final model since accuracy decreases as the number of training epoch increases.

Our study has several shortcomings. First, it is worth mentioning that several models have the problem of overfitting gradient descent after sufficient training; in other words, the model’s prediction is highly consistent with the training set, making it difficult to accurately detect the new data. Therefore, we retained the results when the training reached the highest accuracy; later updates will only consider the original parameters after the original ideal recognition performance is achieved. Second, although we employed transfer learning to improve the accuracy of the ResNet model in identifying gingivitis from oral photographs, our dataset collected from patients was limited; thus, we plan to expand the amount of data from patients of different ages and for a wider range of ethnic groups. Third, some issues during the training process may lead to the failure of gingivitis detection; for instance, improper lighting can cause light spots on gingiva, which may be misidentified as gingivitis (see Fig. 8). Thus, we need composite images with fewer light spots, and pre-processing steps should be designed to reduce noise without eliminating real features. Finally, to improve the model accuracy and reliability, some text datasets such as clinically diagnosed cases or traditional questionnaires [25] can be used to complement image datasets [26].

**Fig. 14:**
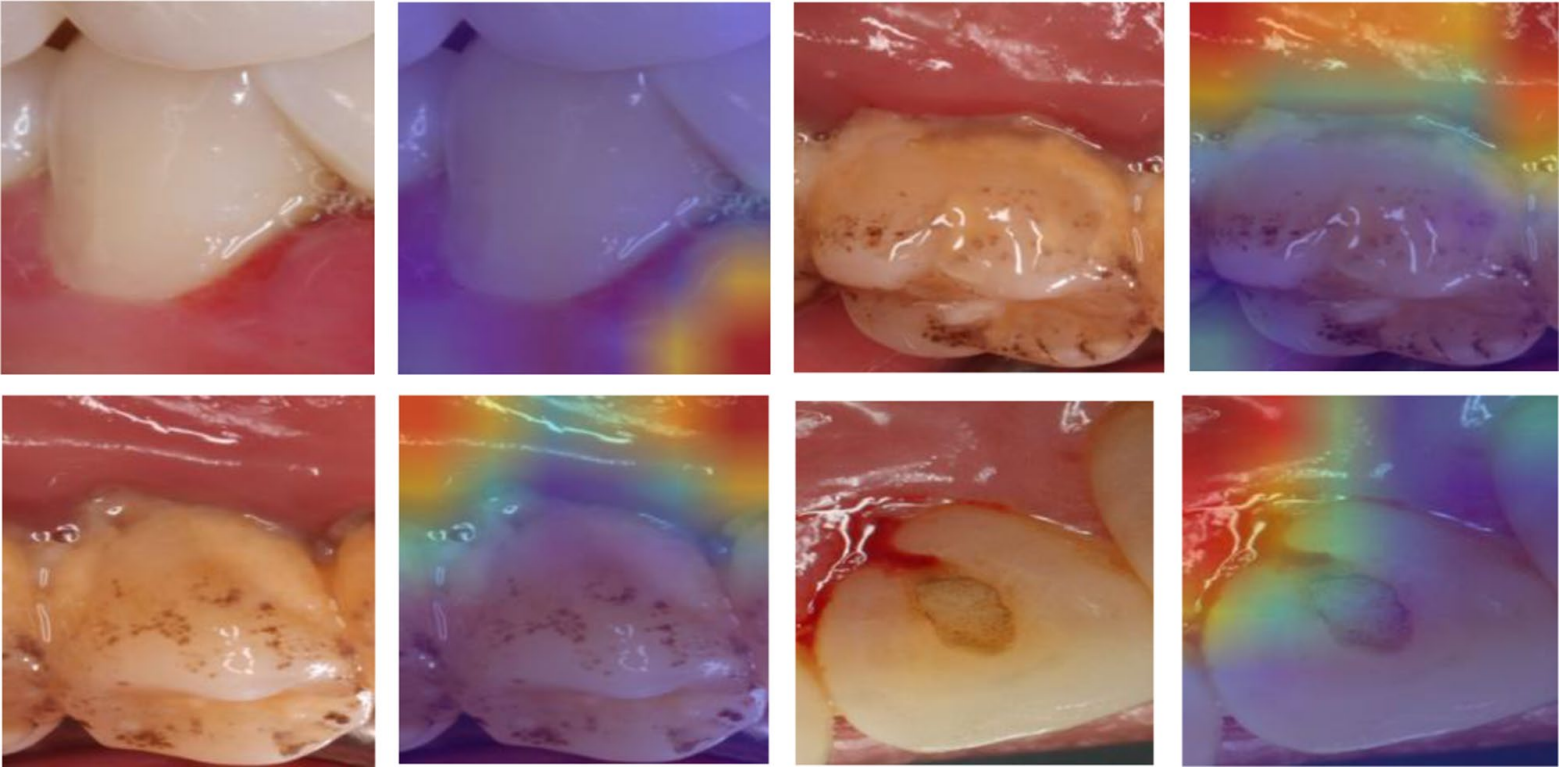
Grad-CAM of correct predictions of gingivitis

**Fig. 15:**
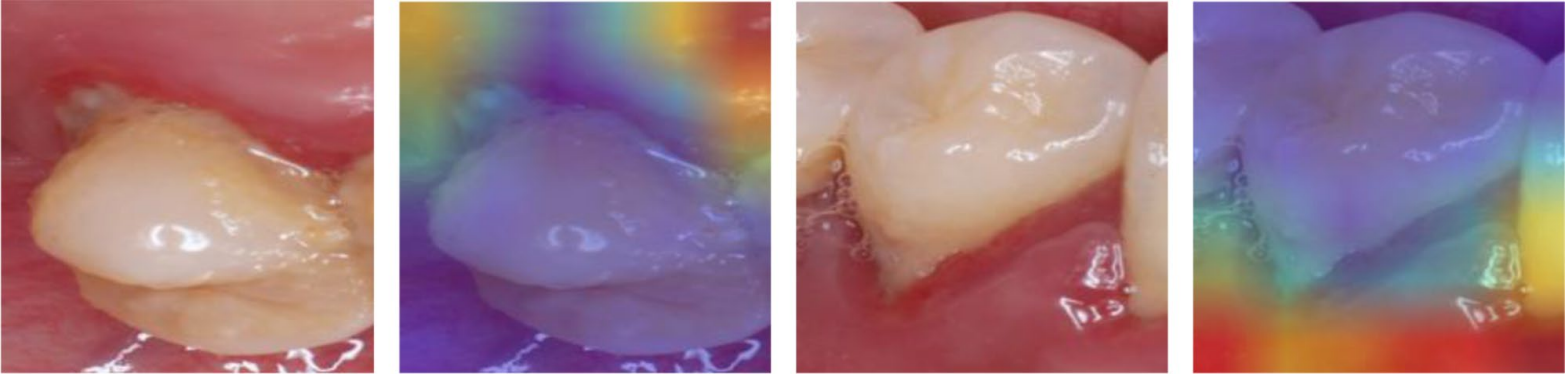
Grad-CAM of incorrect predictions of gingivitis

We show Grad-CAM based on the last convolutional layer of each model on several test data samples. These heatmaps can help understand which areas of the image the model focuses on (Fig. 14). Simultaneously, the incorrect predictions of the picture by the model is likely attributed to issues with reflective light during the shooting process. For instance, the subsequent heat map failed to accurately detect gingivitis. Figure 15 is a heat map illustrating a selection of the images.

## Conclusion

Deep learning plays an essential role in dental disease recognition. We extracted data multiple times for complex tasks with limited data and used ensemble learning to improve model performance. Among the tested models, the *ResNet* and *GoogleNet* models performed best, and transfer learning increased the accuracy of the models to recognize gingivitis from oral images.

## Data Availability

The data used in current study were collected from Medical School of Nanjing University and is available only for the granted research. However, the data can be made available if requested within data protection and regulation guideline. https://github.com/Eating-GET/Tooth_Data. The datasets used during the study will be available from the corresponding author upon request.
